# *LINC00173* facilitates tumor progression by stimulating RAB1B‐mediated PA2G4 and SDF4 secretion in nasopharyngeal carcinoma

**DOI:** 10.1002/1878-0261.13375

**Published:** 2023-01-23

**Authors:** Shi‐Wei He, Ye‐Lin Liang, Yuan Zhang, Xu Liu, Sha Gong, Ming‐Liang Ye, Sheng‐Yan Huang, Xi‐Rong Tan, Shi‐Qing Zhou, Yin Zhao, Na Liu, Ying‐Qing Li

**Affiliations:** ^1^ State Key Laboratory of Oncology in South China, Collaborative Innovation Center of Cancer Medicine, Guangdong Key Laboratory of Nasopharyngeal Carcinoma Diagnosis and Therapy Sun Yat‐sen University Cancer Center Guangzhou China

**Keywords:** *LINC00173*, nasopharyngeal carcinoma, PA2G4, RAB1B, SDF4

## Abstract

An increasing number of studies have found that long non‐coding RNA (lncRNA) play important roles in driving the progression of nasopharyngeal carcinoma (NPC). Our microarray screening revealed that expression of the lncRNA long intergenic non‐protein coding RNA 173 (*LINC00173*) was upregulated in NPC. However, its role and mechanism in NPC have not yet been elucidated. In this study, we demonstrate that high *LINC00173* expression indicated a poor prognosis in NPC patients. Knockdown of *LINC00173* significantly inhibited NPC cell proliferation, migration and invasion *in vitro*. Mechanistically, *LINC00173* interacted and colocalized with Ras‐related protein Rab‐1B (RAB1B) in the cytoplasm, but the modulation of *LINC00173* expression did not affect the expression of RAB1B at either the mRNA or protein levels. Instead, relying on the stimulation of RAB1B, *LINC00173* could facilitate the extracellular secretion of proliferation‐associated 2G4 (PA2G4) and stromal cell‐derived factor 4 (SDF4; also known as 45‐kDa calcium‐binding protein) proteins, and knockdown of these proteins could reverse the NPC aggressive phenotype induced by *LINC00173* overexpression. Moreover, *in vivo LINC00173*‐knockdown models exhibited a marked slowdown in tumor growth and a significant reduction in lymph node and lung metastases. In summary, *LINC00173* serves as a crucial driver for NPC progression, and the *LINC00173*–RAB1B–PA2G4/SDF4 axis might provide a potential therapeutic target for NPC patients.

## Graphical abstract

Our study shows that *LINC00173* is upregulated in nasopharyngeal carcinoma (NPC) and is associated with poor prognosis of patients. *LINC00173* directly binds and interacts with RAB1B, subsequently facilitates PA2G4 and SDF4 secretion through exocytosis pathway, finally, promotes NPC cell proliferation, migration, invasion and metastasis. The *LINC00173*–RAB1B–PA2G4/SDF4 axis might provide a potential therapeutic target for NPC patients.

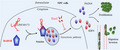

**Abbreviations**
AS: antisense sequences
FISH: immunofluorescence and *in situ* hybridization
GO: gene ontology
IHC: immunohistochemistry
KSFM: keratinocyte serum‐free medium
*LINC00173*
: long intergenic non‐protein coding RNA 173
lncRNA: long non‐coding RNA
MS: mass spectrometry.
NPC: nasopharyngeal carcinoma
PA2G4: proliferation‐associated 2G4
RAB1B: Ras‐related protein Rab‐1B
RIP: RNA immunoprecipitation
SDF4: stromal cell derived factor 4
SS: sense sequences
TNM: tumor node metastasis

## 1 Introduction

Nasopharyngeal carcinoma (NPC) is particularly prevalent in South China, South‐Eastern Asia and North Africa [1, 2]. In 2018, the cases of NPC patients reached 130 000 worldwide and 50% of cases came from China [3]. More than 70% of patients are initially diagnosed with locally advanced disease [1]. In the past decades, the combination of intensity‐modulated radiation therapy and chemotherapy has greatly improved the survival rate of NPC [4, 5, 6]. Nevertheless, about 20% of NPC patients suffer from distant metastasis and/or local recurrence after radical therapy [4, 5, 6]. The occurrence and progression of NPC should be a pathological ecosystem of ‘ecological and evolutionary unity‘ in multidimensional time and space [7]. Therefore, there is an urgent need to explore the mechanisms of NPC progression to identify effective therapeutic targets for NPC patients.

Long non‐coding RNA (lncRNA) are a class of RNA molecules that are longer than 200 nucleotides without protein‐coding potential [8, 9]. They can function as signals, decoys, guides or scaffolds of other biological molecules to constitute a huge and fine regulatory system [10, 11]. Increasing evidence has demonstrated that lncRNA play essential roles in regulating various types of biological functions, and its dysregulation can initiate and promote the carcinogenesis and development of various types of cancers, including NPC [12, 13]. Recently, it has been reported that several dysregulated lncRNA play important roles in regulating NPC proliferation, apoptosis, invasion and metastasis, such as *LINC00930*, *PVT1*, *DANCR*, *FAM225A* and *DIAPH1‐AS1* [14, 15, 16, 17, 18]. The dysregulated lncRNA have also been reported to affect treatment efficacy by regulating NPC cell chemoresistance and radioresistance [19, 20]. A recent study has established an lncRNA signature associated with tumor immune heterogeneity for predicting the metastasis and prognosis of NPC patients [21]. Obviously, the dysregulated lncRNA exert vital roles in NPC carcinogenesis and progression, thus, further studies regarding their regulatory mechanisms will provide novel treatment targets for NPC.

In this study, based on our previous lncRNA profiling [17], we found that *LINC00173* was significantly upregulated in NPC tissues compared with normal nasopharynx tissues. However, the function and mechanism of *LINC00173* remain uncertain in NPC. Clinical sample analysis showed that *LINC00173* was upregulated and correlated with poor prognosis in NPC patients. *In vitro* and *in vivo* functional studies demonstrated that *LINC00173* could promote NPC cell proliferation, migration, invasion and metastasis. Mechanistically, *LINC00173* directly binded to and interacted with RAB1B, thus facilitating PA2G4 and SDF4 secretion in a RAB1B‐mediated manner. Knockdown of PA2G4 or SDF4 was able to reverse *LINC00173*‐mediated NPC progression. Our study elucidates the clinical significance and regulatory mechanism of *LINC00173* in NPC and provides potential therapeutic targets for NPC patients.

## 2 Materials and methods

### 2.1 Clinical samples

We gathered 16 normal nasopharynx tissues and 20 freshly frozen NPC tissues, as well as a set of 214 paraffin‐embedded NPC specimens from the Sun Yat‐sen University Cancer Center (January 2006 to December 2009, Guangzhou, China). All methods and strategies involving human samples were implemented in accordance with the guidelines of the Institutional Ethical Review Boards of Sun Yat‐sen University Cancer Center (GZR2017‐079), and the standards set by the Declaration of Helsinki. This retrospective study was conducted after all patients had completed the visit and the data were anonymous; thus, the requirement of informed consent was waived by the ethics review boards.

### 2.2 Cell culture

Human immortalized normal nasopharyngeal epithelial cell line NP69 (RRID: CVCL_F755) was cultured in KSFM medium (Gibco, Grand Island, NY, USA). Human NPC cell lines, HK1 (RRID: CVCL_7084), SUNE1 (RRID: CVCL_6946), CNE1 (RRID: CVCL_6888), CNE2 (RRID: CVCL_6889), C666‐1 (RRID: CVCL_7949), HNE1 (RRID: CVCL_0308), HONE1 (RRID: CVCL_8706), 5‐8F (RRID: CVCL‐C528) and 6‐10B (RRID: CVCL_C529), were cultured in RPMI 1640 medium (Gibco) complemented with 10% FBS (Gibco); S18 (RRID: CVCL_B0U9), S26 (RRID: CVCL_B0UB) were cultured in DMEM medium (Gibco) containing 10% FBS. NP69 and all NPC cell lines, which had been authenticated, were generously provided by Dr. Mu‐sheng Zeng (Sun Yat‐sen University Cancer Center). The human kidney epithelial cell line HEK293T (RRID: CVCL_0063) were obtained from ATCC (Manassas, VA, USA) and cultured in DMEM medium containing 10% FBS. All cell lines were incubated at 37 °C constant temperature incubator with 5% CO_2_. All cell lines were authenticated using short‐tandem repeat profiling, tested for mycoplasma contamination, and cultured for less than 2 months.

### 2.3 Reverse transcription and quantitative PCR (RT‐qPCR)

Total RNA was isolated from paraffin‐embedded tissues using the QIAGEN FFPE RNeasy kit (Qiagen GmbH, Hilden, Germany), as well as from freshly frozen tissues and cells using the TRIzol Reagent (Life Technologies, Carlsbad, CA, USA). Separation and purification of cell nuclear and cytoplasmic RNA were conducted using the NE‐PER Nuclear and Cytoplasmic Extraction Reagents (Invitrogen, Grand Island, NY, USA). Reverse transcription was performed using the reverse transcriptase (Promega, Madison, WI, USA), and quantitative PCR reactions were conducted using the SYBR® Select Master Mix (Invitrogen). All operations are carried out following the manufacturer's instructions, and the sequences of the primers are listed in the Table S1.

### 2.4 Plasmid construction

Specific shRNA sequences against *LINC00173*, *PA24G* and *SDF4* were designed using the BLOCK‐iT™ RNAi Designer (http://rnaidesigner.thermofisher.com), synthesized by Tsingke Biotechnology Co., Ltd (Beijing, China) and then cloned into the pLKO.1 plasmid (Addgene, Cambridge, MA, USA). The full‐length sequences of *LINC00173* transcript (Ensemble Transcript ID: ENST00000470091; 435 nt) and the CDS sequences of *RAB1B* and *GAPDH* were amplified using Q5® Hot Start High‐Fidelity 2X Master Mix (New England Biolabs, Inc., Ipswich, MA, USA) and cloned into the pHAGE‐6tag‐puro vector (Addgene). All primers are listed in Table S1.

### 2.5 Transfection and stable cell line construction

HK1 and SUNE1 cells were transiently transfected with the indicated plasmids using the Neofect™ DNA transfection reagent (Neofect Tech, Beijing, China) and the transfected cells were harvested for further assays. For stable transfection, HEK293T cells were cotransfected with scramble control (shCtrl) or sh*LINC00173* (sh0173) plasmid with the lentivirus packaging plasmids psPAX2 and pMD2G plasmids (Addgene) using the polyethylenimine (PEI; Sigma‐Aldrich, St. Louis, MO, USA). After 48 h of culture, the supernatant virus liquid was collected by centrifugation and filtered with 0.22 μm Millex‐GP Syringe Filter Unit (Merck Millipore, Billerica, MA, USA), which was used to infect HK1 and SUNE1 cells, and the stably infected cells were selected and maintained with Puromycin (Gibco).

### 2.6 CCK‐8 and colony formation

For the CCK‐8 assay, 1 × 10^3^ cells were seeded in 96‐well plate (NEST Biotechnology, Wuxi, China), 10 μL CCK‐8 reagent was added and incubated for 2 h, and the OD 450 value was measured at the gradient time (1, 2, 3, 4 and 5 days). The cell growth curves were drawn using graphpad prism (GraphPad Prism, San Diego, CA, USA). For the colony formation assay, 600 cells were planted into the 6‐well plate (NEST Biotechnology). Cell colonies were fixed, stained and counted.

### 2.7 Transwell assays

Transfected HK1 or SUNE1 cells were resuspended in serum‐free medium and seeded into the upper Transwell Chamber (Costar, Cambridge, MA, USA). To the lower Chamber was added 500 μL medium containing 15% FBS, was incubated for 12 or 24 h. The chambers of invasion and migration assays were covered with or without Matrigel (BD Biosciences, San Diego, CA, USA). The migrated and invaded cells were fixed, stained and counted.

### 2.8 RNA pull‐down, mass spectrometry (MS)

The sense and anti‐sense chains of *LINC00173* were transcribed using the MEGAscript™ T7 Transcription Kit (Thermo Fisher Scientific, Waltham, MA, USA) and then labeled with biotin using the Pierce™ RNA 3′ End Desthiobiotinylation Kit (Thermo Fisher Scientific). The pull‐down of incubating biotin‐labeled RNA and cell lysates was conducted using the Magnetic RNA‐Protein Pull‐Down Kit (Thermo Fisher Scientific) in accordance with the manufacturer's instructions. The pulled‐down proteins were subjected to mass spectrometry analysis (FitGene Biotechnology, Guangzhou, China). Differentially enriched proteins were subjected to Gene Ontology (GO) analyses using david software (https://david.ncifcrf.gov/).

### 2.9 RNA immunoprecipitation (RIP)

HK1 or SUNE1 cell lysates were incubated with 2 μg anti‐RAB1B antibody (ABclonal, Wuhan, China) or 2 μg anti‐IgG (Sigma‐Aldrich) at 4 °C for 2 h, then Protein A/G Plus Agarose (Santa Cruz Biotechnology, Santa Cruz, CA, USA) was added and the lysates rotated at room temperature for 1 h. The immunoprecipitated RNA were purified and detected by RT‐qPCR assay. Primers are listed in Table S1.

### 2.10 Immunofluorescence and *in situ* hybridization (FISH)

The Cy3‐tagged *LINC00173* FISH probe was purchased from Ribo Bio (Guangzhou, China). HK1 and SUNE1 cells were fixed and bound with the *LINC00173* probe at 42 °C for 12~16 h, and then incubated with anti‐RAB1B antibody at 4 °C overnight. Finally, the cells were co‐incubated with fluorescent secondary anti‐IgG antibody (Life Technologies) with Hoechst 33342 (Invitrogen) for nucleus staining. The fluorescence pictures were captured using a NIKON ECLIPSE Ni‐U confocal microscope (Nikon, Tokyo, Japan).

### 2.11 Western blotting analysis

Total protein was extracted using the RIPA lysis buffer (Beyotime Biotechnology Inc., Shanghai, China) and quantified using the BCA reagent (Thermo Fisher Scientific). The denatured proteins were subjected to SDS/PAGE gel and transferred to PVDF membrane (Merck Millipore). The membrane was cut according to protein size and incubated with primary antibody: anti‐RAB1B (1 : 1000, ABclonal), anti‐PA2G4 (1 : 1000, ABclonal), anti‐SDF4 (1 : 1000, ABclonal), anti‐Flag (1 : 2000; Sigma‐Aldrich) or anti‐Tubulin (1 : 5000; Woburn, MA, USA). After washing with TBST solution, the bands were incubated with peroxidase‐conjugated goat anti‐mouse or anti‐rabbit IgG antibody (Proteintech, Wuhan, China) and the protein bands were developed using a chemiluminescence instrument (Bio‐Rad, Hercules, CA, USA).

### 2.12 Mass spectrometry analysis for concentrated supernatant

Stable *LINC00173* knockdown (sh0173) or scramble control (shCtrl) SUNE1 cells were cultured to logarithmic growth phase and then replaced with serum‐free medium when the cell confluence reached 80–90%. After being continually cultivated for 16 h, cell culture supernatants were concentrated using Amicon Ultra Tube (Merck Millipore) and accurately measured. Finally, the denatured concentrated supernatants were subjected to SDS/PAGE and LC–MS/MS.

### 2.13 Exo‐1 treatment

Exo‐1 is a chemical inhibitor of the exocytic pathway [22]. After being transfected with the *LINC00173* overexpression plasmid, HK1 and SUNE1 cells were treated with 20 μm Exo‐1 (TargetMol, Wellesley Hills, MA, USA) for 24 h, and the cells then digested for CCK‐8, colony formation, Transwell assays and western blotting assays.

### 2.14 *In vivo* tumor xenograft models

All animal experiments were approved by the Institutional Animal Care and Use Ethics Committee of Sun Yat‐sen University Cancer Center (No. L102012019120J). Female BALB/c nude mice (4~5 weeks old) were purchased from the Charles River Laboratories (Beijing, China). Animal care was conducted in the specific pathogen‐free barrier facility at Sun Yat‐sen University Cancer Center as required by the guidelines of the Animal Care and Use Committee. For tumor growth model, 1 × 10^6^ SUNE1 cells stably expressing shCtrl or sh0173 were injected into the underarm skin of mice (*n* = 8 per group) and the tumor size was measured twice a week. After 30 days, the tumors were dissected and weighed. Total RNA of tumors was isolated for the detection of *LINC00173*. Tumor sections were subjected to immunohistochemistry (IHC) analysis for RAB1B, PA2G4 and SDF4 levels.

For the inguinal lymph node metastasis model, 3 × 10^6^ SUNE1 cells stably expressing sh0173 or shCtrl were injected into the foot pad of mice (*n* = 8 per group). After 40 days, the foot pad tumors and inguinal lymph nodes were detached from sacrificed mice. The paraffin sections of foot pad tumors were stained using H&E (Solarbio, Beijing, China) and the paraffin sections of inguinal lymph nodes were subjected to IHC with anti‐pan‐cytokeratin antibody (Thermo Fisher Scientific). For the lung metastasis model, 1 × 10^6^ SUNE1 cells stably expressing sh0173 or shCtrl were injected into the tail vein of mice (*n* = 6 per group). After approximately 2 months tumor bearing, lung tissues were taken out from the thoracic cavity of sacrificed nude mice, and the paraffin sections of the lung were stained using H&E staining.

### 2.15 Immunohistochemistry

The immunohistochemistry assay was performed as previously described [23]. Briefly, sections were deparaffinized and rehydrated, antigen retrieved using sodium citrate solution (Beyotime), blocked with Immunofluorescence blocking solution (Beyotime), then incubated with primary antibody at 4 °C overnight and HRP‐labeled rabbit or mouse second antibodies (Boster, Wuhan, China). Finally, the sections were stained using DAB reagent kit (Beyotime). Images were captured with an upright immunohistochemistry microscope NIKON Eclipse Ni‐U (Nikon). The primary antibodies are as follow: RAB1B (ABclonal; 1 : 200), PA2G4 (ABclonal; 1 : 100) and SDF4 (ABclonal; 1 : 100).

### 2.16 Statistical analyses

All statistical analyses were performed using the spss 22.0 software (IBM, Chicago, IL, USA) and all data are shown as the mean ± SD. Comparison of differences between groups were conducted using Student's *t*‐test and chi‐square test. Survival curves were generated using the Kaplan–Meier method and differences were analyzed with the log‐rank test. The independent prognostic factors were tested using univariate and multivariate Cox regression analysis. *P*‐values less than 0.05 were considered statistically significant.

## 3 Results

### 3.1 *LINC00173* is upregulated and correlates with poor prognosis in NPC

**Fig. 1:**
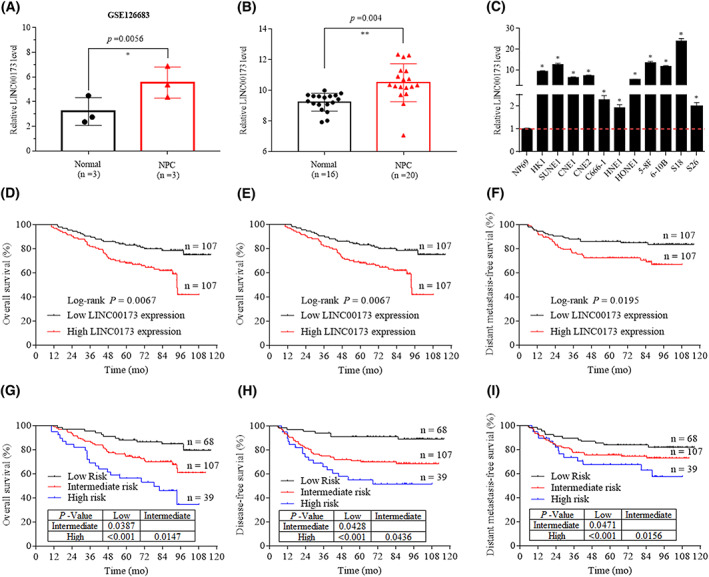
*LINC00173* is upregulated and related to poor prognosis in nasopharyngeal carcinoma (NPC). (A) *LINC00173* expression in NPC from GEO Dataset (GSE126683, normal = 3 cases, NPC = 3 cases). (B) *LINC00173* expression in freshly frozen NPC tissues (*n* = 20) and normal nasopharynx tissues (*n* = 16). (C) *LINC00173* expression in NPC cell lines and normal nasopharyngeal epithelial cell line NP69 (*n* = 3). (D–F) Kaplan–Meier analysis of overall survival (D), disease‐free survival (E) and distant metastasis‐free survival (F) in NPC patients (high *LINC00173* expression, *n* = 107; low *LINC00173* expression, *n* = 107). Relative *LINC00173* expression in NPC tissues of 214 patients were measured by RT‐qPCR assay. High and low *LINC00173* expression groups were divided using the median expression value. (G–I) Kaplan–Meier analysis of overall survival (G), disease‐free survival (H) and distant metastasis‐free survival (I) according to the prognostic prediction model, low risk [early tumor‐node‐metastasis (TNM) stage and low *LINC00173* expression, *n* = 68], intermediate risk (advanced TNM stage or high *LINC00173* expression, *n* = 107) and high risk groups (advanced TNM stage and high *LINC00173* expression, *n* = 39). The data of (A–C) were shown as mean ± SD, and the *P*‐values were determined by Student's *t*‐test (*P < 0.05; **P < 0.01). The data of (D–I) were measured by the Kaplan–Meier method, and the *P*‐values were determined by the log‐rank test.

Recent evidence shows that lncRNA are dysregulated in NPC and play important roles in regulating malignant phenotype [14, 15, 16, 17, 18]. We analyzed the lncRNA expression between three pairs of NPC and normal nasopharynx tissues based on our previous microarray data (GSE126683) [17]. Employing differential analyses, we screened out *LINC00173* (Ensembl Transcript ID: ENST00000470091), which was significantly upregulated in NPC tissues compared with normal nasopharynx tissues (Fig. 1A). For verification, we examined the expression of *LINC00173* in freshly frozen tissues and found that *LINC00173* had significantly higher expression in NPC tissues (Fig. 1B). Moreover, the expression of *LINC00173* in NPC cell lines were remarkably higher than that in normal nasopharyngeal epithelial cell line NP69 (Fig. 1C).

To investigate the clinical significance of *LINC00173* dysregulation in NPC patients, we performed RT‐qPCR assays of total RNA from a cohort of 214 NPC samples. The patients were divided into a high‐expression group (*n* = 107) and a low‐expression group (*n* = 107) based on the median expression level of *LINC00173*. No significant correlations were found between the *LINC00173* expression and clinicopathologic features of NPC patients (Table S2). However, Kaplan–Meier survival analysis showed that, compared with NPC patients with low *LINC00173* expression, those with high *LINC00173* expression had poorer overall survival, disease‐free survival and distant metastasis‐free survival (Fig. 1D–F).

Then, we performed univariate and multivariate Cox regression analysis and found that the *LINC00173* expression level and the TNM stage were significant and independent prognostic determinants (Table S3). Therefore, we combined the *LINC00173* expression level with the TNM stage to construct a comprehensive prognostic model, which classified NPC patients into the following three groups: a high‐risk group with high *LINC00173* expression and advanced TNM stage; an intermediate‐risk group with high *LINC00173* expression or advanced TNM stage; and a low‐risk group with low *LINC00173* expression and early TNM stage. Kaplan–Meier curves analysis revealed significant differences in overall survival, disease‐free survival and distant metastasis‐free survival among these groups, which further confirmed the prognostic value of *LINC00173* in NPC (Fig. 1G–I).

### 3.2 *LINC00173* promotes NPC cell proliferation, migration and invasion *in vitro*

To verify whether *LINC00173* was a non‐coding RNA, we first used LNCipedia database and PhyloCSF scores to predict the potential coding ability of *LINC00173*. The results showed that *LINC00173* was composed of three exons, its full length was 435 nt, and the PhyloCSF score was −91.86, which identified it as a modestly conserved locus (Fig. S1A,B). The PhyloCSF scores were displayed by six frames for each codon, regions with a score less than 0 were identified as non‐coding regions, whereas a region with a score greater than 0 was predicted to be a coding region (Fig. S1C). To further validate this result, the full length of *LINC00173* was cloned into Flag‐tag plasmid and transfected into 293T cells (Fig. S1D). Western blotting with anti‐Flag antibody was used to detect the protein band of each group. The results displayed no protein expression in *LINC00173*‐transfected cells, suggesting that *LINC00173* had no peptide or protein coding ability (Fig. S1E).

**Fig. 2:**
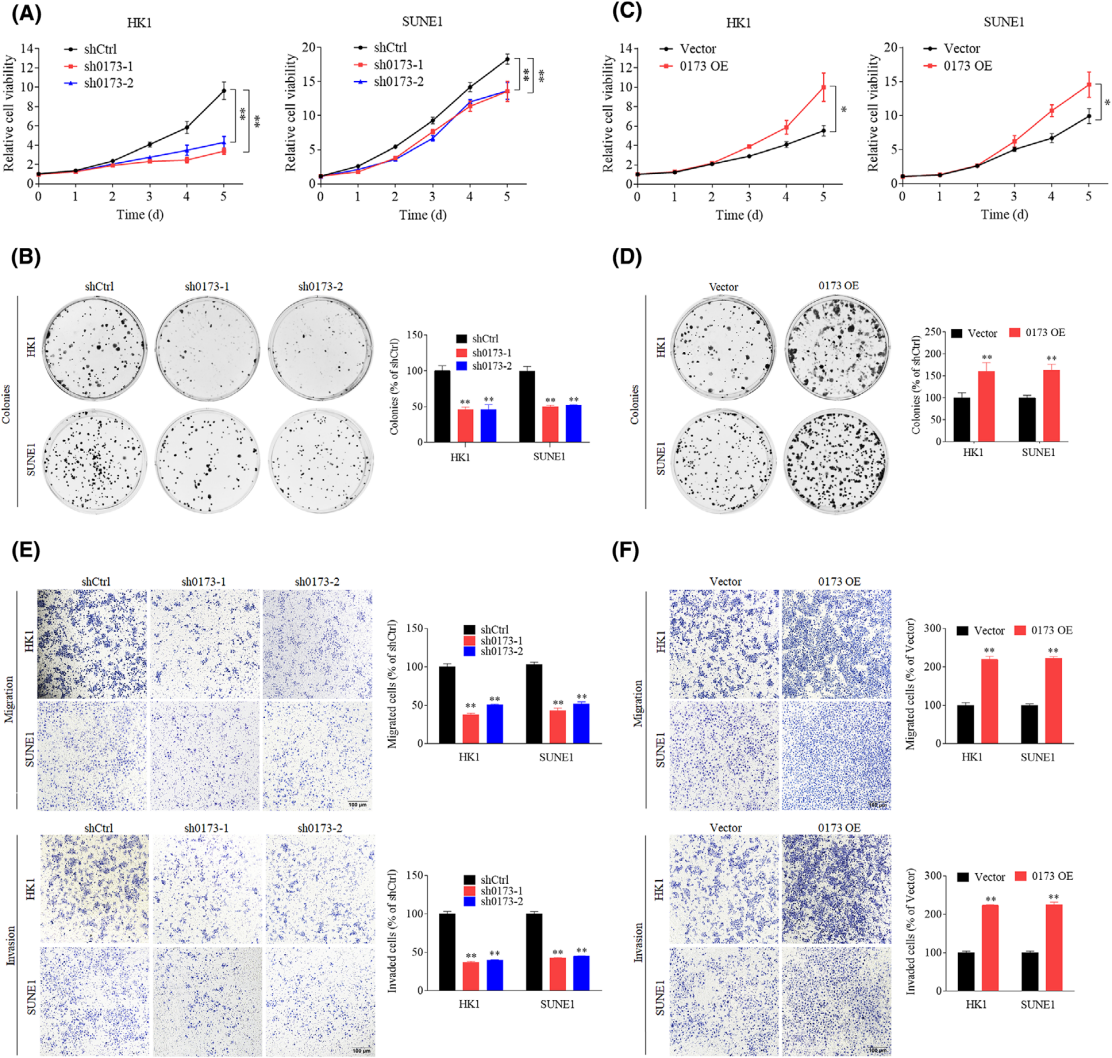
*LINC00173* promotes nasopharyngeal carcinoma (NPC) cells proliferation, migration and invasion *in vitro*. (A,B) Cell proliferation curves (A) and clonogenic assays (B) of HK1 and SUNE1 cells transfected with shCtrl or sh*LIN00173* (sh0173) plasmid (*n* = 3). (C,D) Cell proliferation curves (C) and clonogenic assays (D) of HK1 and SUNE1 cells transfected with vector or *LIN00173* overexpression (OE) plasmid (*n* = 3). (E) Transwell migration and invasion assays of HK1 and SUNE1 cells transfected with shCtrl or sh0173 vector plasmids (*n* = 3). Scale bar: 100 μm. The migrated or invaded cells were counted. (F) Transwell migration and invasion assays of HK1 and SUNE1 cells transfected with vector or *LIN00173* OE plasmids (*n* = 3). Scale bar: 100 μm. Data are presented as mean ± SD. *P*‐values were determined by Student's *t*‐test (*P < 0.05; **P < 0.01).

To clarify the biological functions of *LINC00173* dysregulation in NPC, we first performed CCK8, colony formation, Transwell migration and invasion assays in HK1 and SUNE1 cells after knockdown or overexpression of *LINC00173* (Fig. S2A,B). Knockdown of *LINC00173* severely impeded the cell proliferation and colony formation abilities (Fig. 2A,B), whereas overexpression of *LINC00173* manifested contrary functions (Fig. 2C,D). In addition, knockdown of *LINC00173* remarkably impaired the cell migration and invasion abilities (Fig. 2E), whereas upregulation of *LINC00173* strengthened the cell migration and invasion abilities (Fig. 2F). The above findings demonstrate that *LINC00173* can promote cell proliferation and metastasis in NPC, accounting for its association with poor prognosis.

### 3.3 *LINC00173* directly interacts with RAB1B

**Fig. 3:**
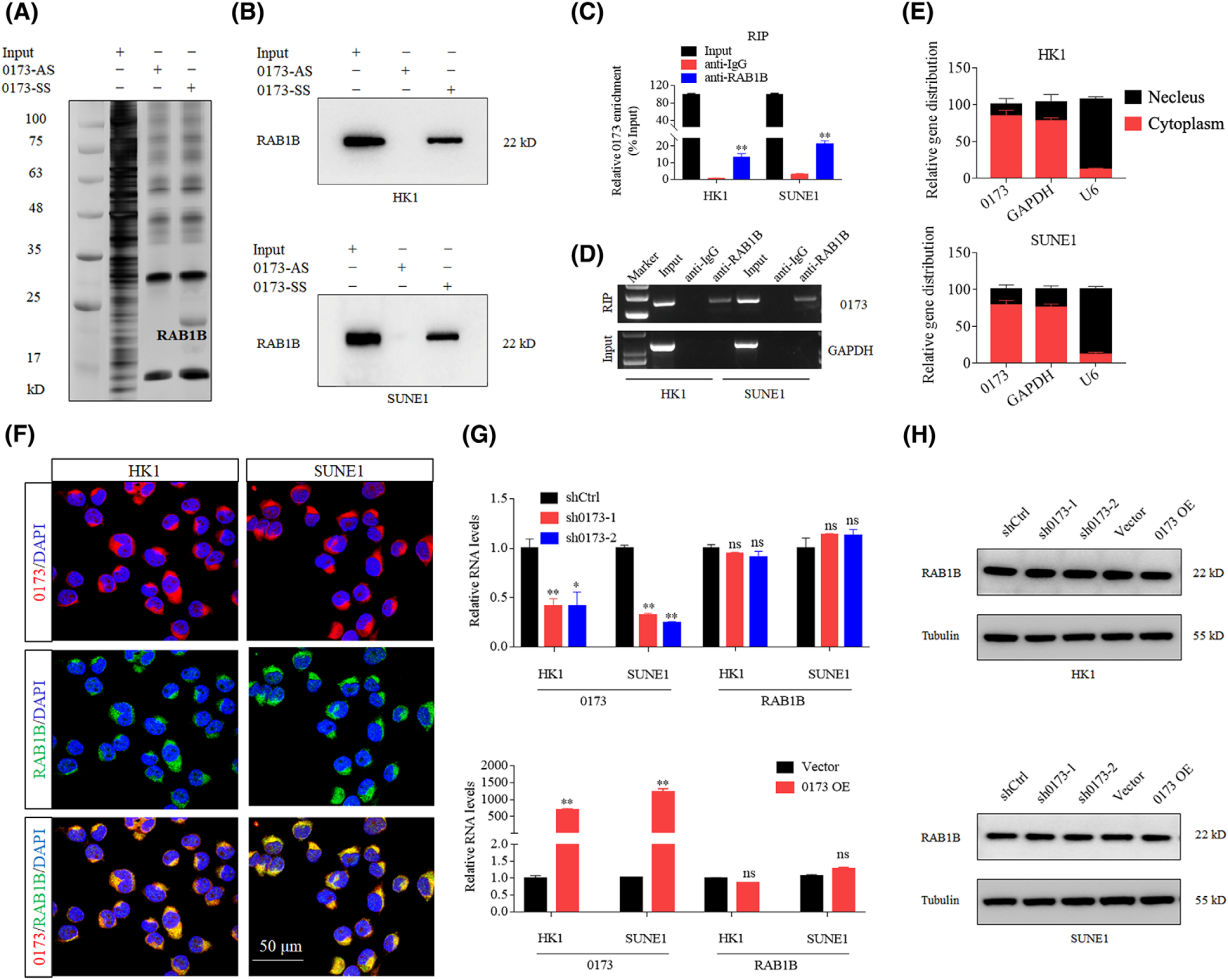
*LINC00173* directly interacts with RAB1B. (A) Biotin‐labeled *LINC00173* sense (SS) and anti‐sense (AS) RNA pulled down samples were subjected to SDS/PAGE electrophoresis and silver stained (*n* = 3). RAB1B is a potential interactive candidate of *LINC00173*. (B) RNA pulldown with western blot (WB) analysis of the interactions of *LINC00173* and RAB1B in HK1 and SUNE1 cells (*n* = 3). (C) RNA immunoprecipitation (RIP) with anti‐RAB1B antibody in HK1 and SUNE1 cells revealed RAB1B protein bound to *LINC00173* RNA. Relative enrichment of *LINC00173* levels interacted with RAB1B proteins was normalized to Input extraction (10% total extraction without immunoprecipitation, *n* = 3). (D) Agarose gel electrophoresis showed the mount of *LINC00173* in the above RIP samples (*n* = 3). GAPDH was used as a negative control. (E) Nucleocytoplasmic separation and RT‐qPCR assays detected *LINC00173* expression in cell nucleus and cytoplasm (*n* = 3). (F) Confocal imaging of *LINC00173* and RAB1B in HK1 and SUNE1 cells was visualized by RNA‐FISH and immunofluorescence assays (*n* = 3). Scale bar: 50 μm. (G) Relative levels of *LINC00173* and RAB1B in HK1 and SUNE1 cells after transfection with sh*LINC00173* or *LINC00173* overexpression plasmids (*n* = 3). (H) Relative levels of RAB1B protein in HK1 and SUNE1 cells after transfection with sh*LINC00173* or *LINC00173* overexpression plasmids (*n* = 3). Data of (C and G) are presented as mean ± SD. *P*‐values were determined by Student's *t*‐test (*P < 0.05; **P < 0.01).

To search out the regulatory mechanism of *LINC00173* in NPC, we performed an RNA pull‐down assay with biotin‐labeled *LINC00173* sense or antisense sequence (0173 SS or AS) followed by mass spectrometry (MS) to seek for specific *LINC00173* interacting proteins. The GO analysis indicated that the *LINC00173* interacting proteins were mainly enriched on the cytoplasm and exosomes in the cellular component aspect, catalytic activity and transporter activity in the molecular function aspect, signal transduction and metabolism in the biological aspect (Table S4, Fig. S3A–C). The silver staining of pulled‐down proteins showed that specific proteins could be detected in 0173 SS group, and MS identified the RAB1B protein for further analysis (Fig. 3A, Table S4). RAB1B is a member of the RAB protein family and is related to exocytic transport of secreted proteins [24, 25]. To further validate the physical association between *LINC00173* and RAB1B, we performed RNA pull‐down assay followed by western blotting with RAB1B antibody. The chemiluminescence results suggested that *LINC00173* highly interacted with RBA1B in HK1 and SUNE1 cells (Fig. 3B). We conducted RIP followed by RT‐qPCR, which indicated that *LINC00173* was obviously enriched by RAB1B antibody (Fig. 3C,D).

To examine the cellular localization of *LINC00173* in NPC cells, we conducted nuclear‐cytoplasmic RNA extraction assays; *LINC00173* was mainly localized in the cytoplasm of HK1 and SUNE1 cells (Fig. 3E). FISH and immunofluorescence (IF) assays showed that *LINC00173* and RAB1B protein were colocalized in the cytoplasm of HK1 and SUNE1 cells (Fig. 3F). Interestingly, neither knockdown nor overexpression of *LINC00173* influenced RAB1B mRNA or protein levels (Fig. 3G,H). Taken together, these findings illustrate that *LINC00173* directly interacts with RAB1B protein in the cytoplasm but might not affect the expression of RAB1B.

### 3.4 *LINC00173* facilitates PA2G4 and SDF4 secretion in a RAB1B‐mediated manner

**Fig. 4:**
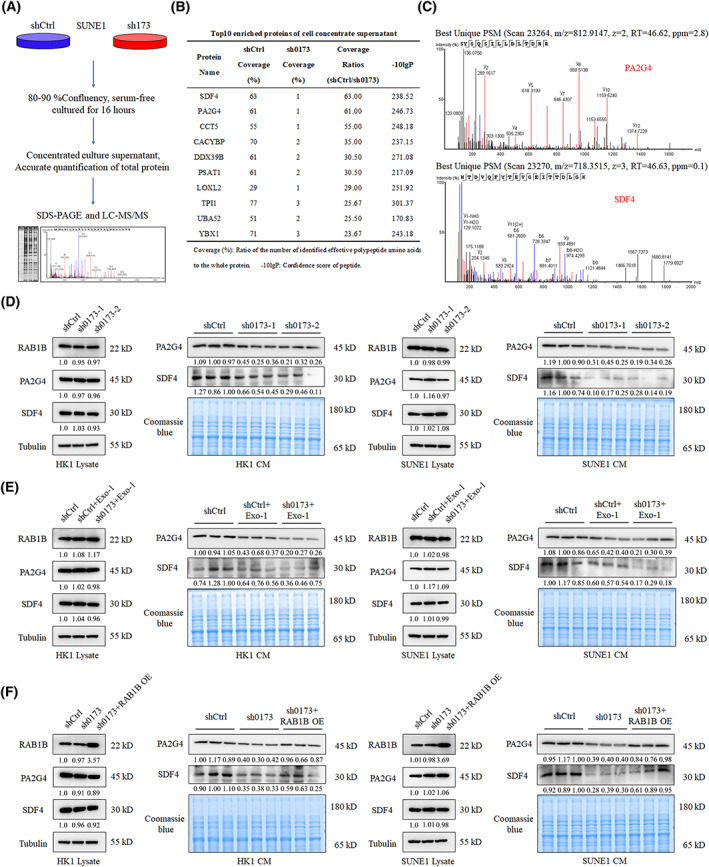
*LINC00173* facilitates PA2G4 and SDF4 secretion in a RAB1B‐mediated manner. (A) Workflows of mass spectrometry (MS) analysis for *LINC00173* regulated specific secreted proteins. (B) Top10 enriched proteins of concentrated supernatants in shCtrl and sh*LINC00173* (sh0173) group. (C) The peptide peak spectrum of PA2G4 and SDF4 proteins according to the MS analysis. (D) Western blot (WB) assay detected the RAB1B, PA2G4 and SDF4 expression in the lysate and concentrated supernatants of HK1 and SUNE1 cells transfected with sh0173 plasmid (*n* = 3). (E) WB assay detected the RAB1B, PA2G4 and SDF4 expression in lysate and concentrated supernatants of HK1 and SUNE1 cells transfected with sh0173 plasmid and treated with Exo‐1 (*n* = 3). (F) WB assay detected the RAB1B, PA2G4 and SDF4 expression in lysate and concentrated supernatants of HK1 and SUNE1 cells cotransfected with sh0173 and RAB1B overexpression (OE) plasmids (*n* = 3). The gels were stained with Coomassie blue to determine the total protein consistent loading in each lane.

As a member of the RAB protein family, RAB1B can regulate the exocytic trafficking of proteins and lipids [24, 25]. Thus, we investigated whether *LINC00173* functioned by regulating the RAB1B‐mediated exocytosis pathway. We performed LC–MS/MS to screen for differentially secreted proteins facilitated by *LINC00173*/RAB1B. SUNE1 cells stably expressing sh0173 or shCtrl were cultured and replaced with the serum‐free medium when the cell confluence reached 80–90%. After being cultured for 16 h, supernatants were concentrated with ultrafiltration tubes and the concentrates were measured and subjected to SDS/PAGE and LC–MS/MS (Fig. 4A). The top 10 differentially secreted proteins were filtered out (Fig. 4B, Table S5), among which PA2G4 and SDF4 were the mostly highly enriched proteins, showing a remarkable decline after *LINC00173* knockdown (Fig. 4C).

To confirm whether the secretion of PA2G4 and SDF4 proteins was mediated by the *LINC00173*/RAB1B, we performed western blotting assays for the detection of RAB1B, PA2G4 and SDF4 in both the cell lysate and concentrated supernatants. After *LINC00173* knockdown, total levels of RAB1B, PA2G4 and SDF4 proteins in cell lysate were unchanged, whereas the levels of PA2G4 and SDF4 were significantly decreased in concentrated supernatants of both the HK1 and SUNE1 cells (Fig. 4D).

To explore whether *LINC00173*/RAB1B regulates the PA2G4 and SDF4 secretion through the exocytic pathway, we first treated NPC cells with Exo‐1, a chemical inhibitor of the exocytic pathway [22]. As the data showed, the levels of PA2G4 and SDF4 proteins severely declined after Exo‐1 treatment in the concentrated supernatants of HK1 and SUNE1 cells (Fig. 4E), demonstrating that *LINC00173*/RAB1B facilitates the PA2G4 and SDF4 secretion via the exocytic pathway. To further investigate whether *LINC00173* promotes PA2G4 and SDF4 protein secretion in a RAB1B‐mediated manner, we transfected RAB1B overexpression plasmid into NPC cells stably expressing sh0173. As expected, overexpression of RAB1B could reverse the secretion of PA2G4 and SDF4 protein in *LINC00173* knockdown HK1 and SUEN1 cells (Fig. 4F).

Jointly, these findings reveal that *LINC00173* can stimulate PA2G4 and SDF4 secretion in a RAB1B‐mediated exocytic pathway.

### 3.5 Knockdown of PA2G4 or SDF4 reverses *LINC00173*‐mediated NPC progression

**Fig. 5:**
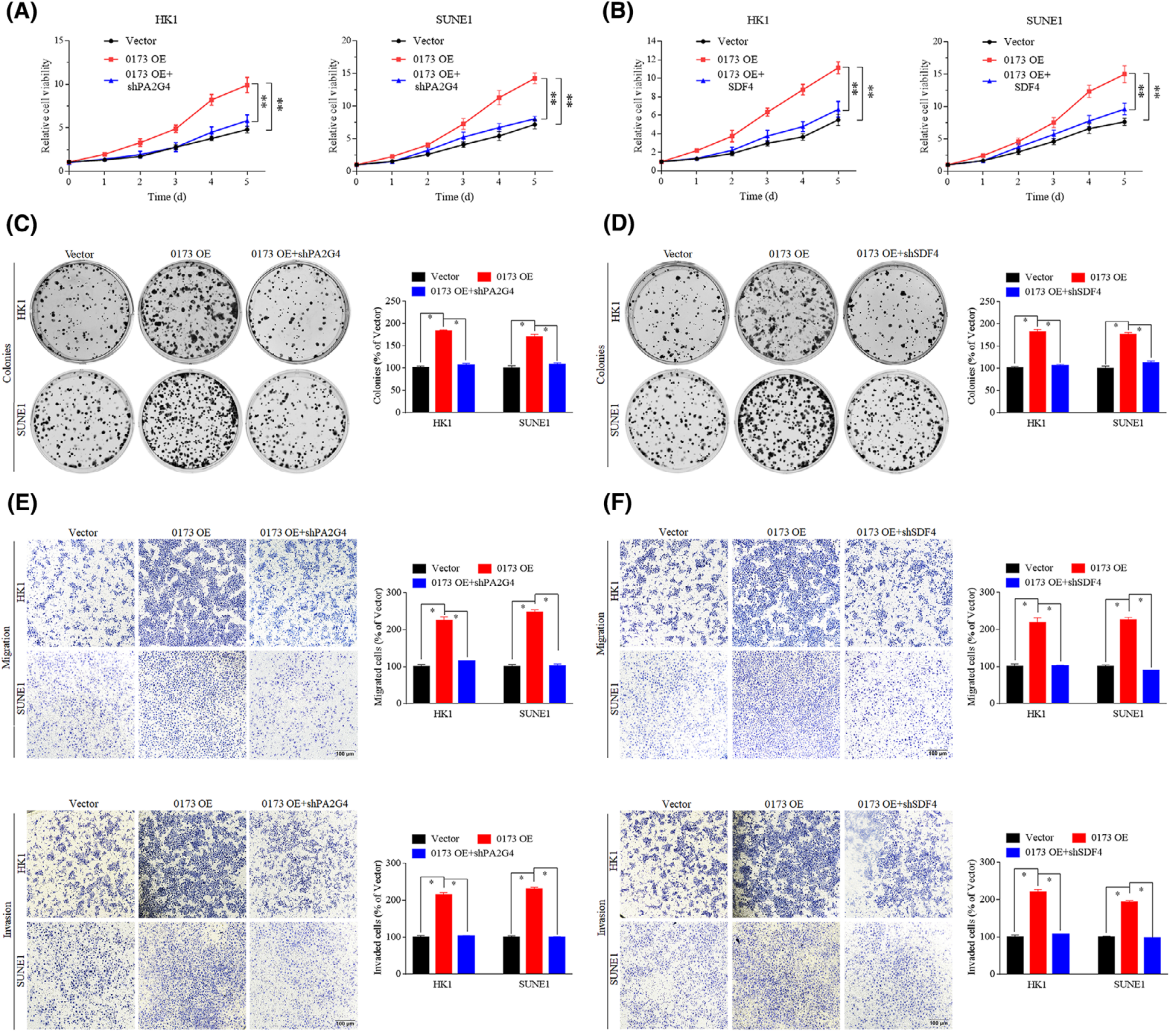
Knockdown of PA2G4 or SDF4 reverses *LINC00173*‐mediated nasopharyngeal carcinoma (NPC) progression. (A) Cell proliferation curves of HK1 and SUNE1 cells cotransfected with *LINC00173* overexpression (0173‐OE) and sh*PA2G4* plasmids (*n* = 3). (B) Cell proliferation curves of HK1 and SUNE1 cells cotransfected with 0173‐OE and sh*SDF4* plasmids (*n* = 3). (C) Clonogenic assays of HK1 and SUNE1 cells cotransfected with 0173‐OE and sh*PA2G4* plasmids (*n* = 3). (D) Clonogenic assays of HK1 and SUNE1 cells cotransfected with 0173‐OE and sh*SDF4* plasmids (*n* = 3). (E) Transwell migration and invasion assays of HK1 and SUNE1 cells cotransfected with 0173‐OE and sh*PA2G4* plasmids (*n* = 3). Scale bar: 100 μm. (F) Transwell migration and invasion assays of HK1 and SUNE1 cells cotransfected with 0173‐OE and sh*SDF4* plasmids (*n* = 3). Scale bar: 100 μm. Data are presented as mean ± SD. *P*‐values were determined by Student's *t*‐test (*P < 0.05; **P < 0.01).

To determine whether *LINC00173*/RAB1B promotes NPC progression by facilitating the secretion of PA2G4 and SDF4 proteins, we cotransfected *LINC00173* overexpression plasmid with PA2G4 or SDF4 shRNA plasmid into NPC cells for the proliferation and invasion assays (Fig. S4). The growth curve and colony formation data showed that knockdown of PA2G4 or SDF4 remarkably reversed the enhancement effect on cell growth and proliferation induced by *LINC00173* overexpression (Fig. 5A–D). Moreover, overexpression of *LINC00173* significantly increased the migrative and invasive abilities of HK1 and SUNE1 cells, whereas knockdown of PA2G4 or SDF4 was able to reverse the above aggressive phenotypes (Fig. 5E,F). Collectively, our data suggest that *LINC00173* can promote NPC cell proliferation, migration and invasion via facilitating PA2G4 and SDF4 secretion.

### 3.6 Knockdown of *LINC00173* inhibits NPC tumor growth and metastasis *in vivo*

**Fig. 6:**
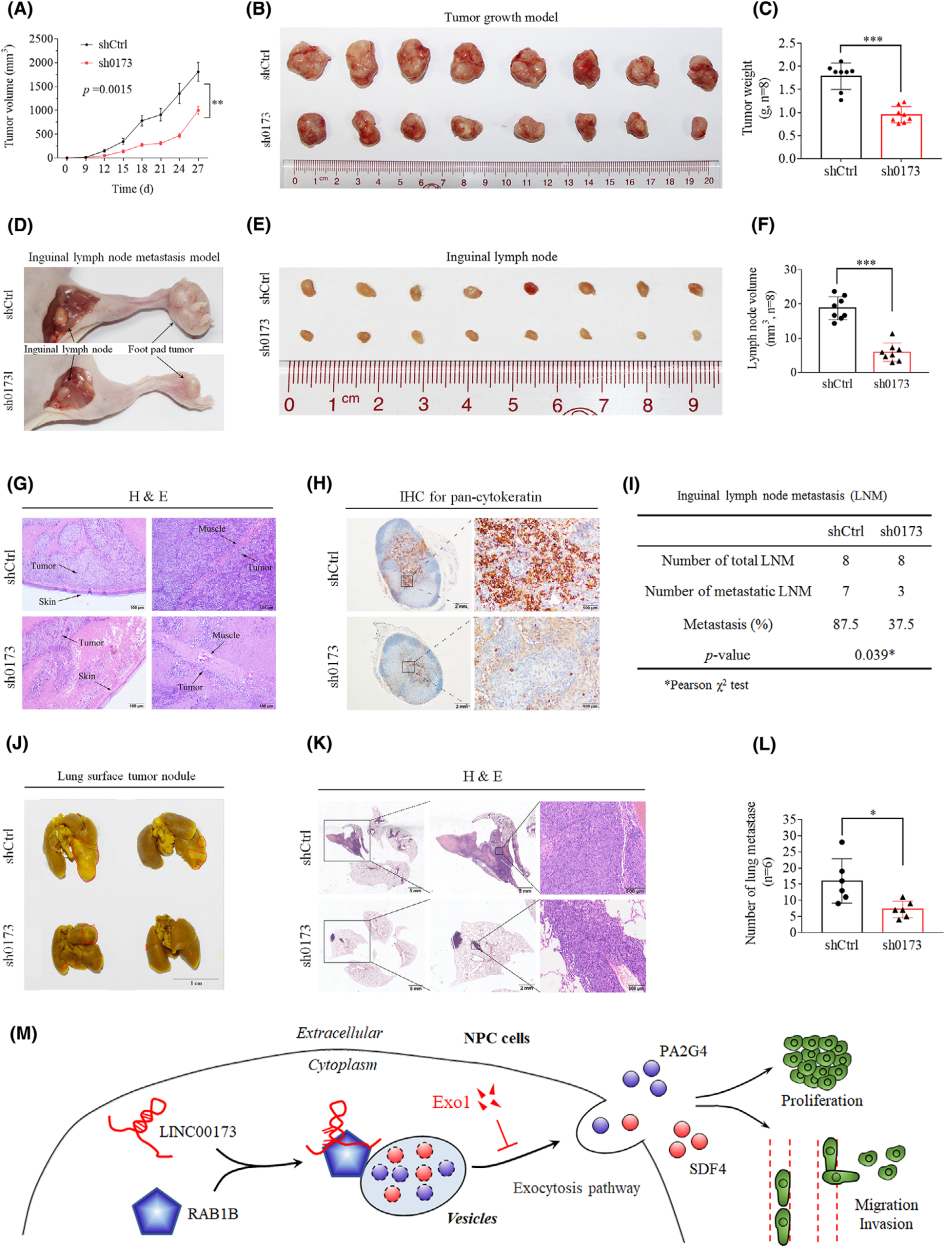
Knockdown of *LINC00173* inhibits nasopharyngeal carcinoma (NPC) tumor growth and metastasis *in vivo*. (A) Tumor growth curves of nude mice injected with stable *LINC00173* knockdown (sh0173) or shCtrl SUNE1 cells (*n* = 8). (B) Representative xenograft tumor images from shCtrl and sh0173 groups (*n* = 8). (C) Average weight of tumors for shCtrl and sh0173 group (*n* = 8). (D) Representative inguinal lymph node and foot pad tumor images of lymphatic metastasis model for shCtrl and sh0173 groups (*n* = 8). (E) Representative inguinal lymph node images for shCtrl and sh0173 groups (*n* = 8). (F) Average volume of inguinal lymph nodes for the sh0173 and shCtrl group (*n* = 8). (G) H&E staining for foot pad primary tumors (*n* = 8). Scale bar: 50 μm. (H) Pan‐cytokeratin staining for the inguinal lymph nodes from both groups (*n* = 8). Scale bars: 2 mm and 500 μm. (I) Pearson's χ^2^ test for metastatic ratio of inguinal lymph nodes in the sh0173 and shCtrl group (*n* = 8). (J) Representative images of metastatic nodules in lung metastasis model (*n* = 8). Scale bar: 1 cm. (K) H&E staining of lung tissues from shCtrl and sh0173 groups (*n* = 8). Scale bars: 5 mm, 2 mm and 500 μm. (L) Average number of metastatic nodules for the sh0173 and shCtrl group (*n* = 8). (M) Graphical abstract of *LINC00173* function and mechanism in NPC. *P*‐values of (A, C, F and L) were determined by Student's *t*‐test; data are presented as mean ± SD (*P < 0.05; **P < 0.01; ***P < 0.001). The *P*‐value of (I) was determined by Pearson's χ^2^ test (*P < 0.05).

To explore whether *LINC00173* promotes the growth of NPC cells *in vivo*, we established subcutaneous tumor models using SUNE1 cells stably expressing sh0173 or shCtrl. Compared with the shCtrl group, the sh0173 group displayed a decreased xenograft growth in terms of the volume, size and weight of the stripped tumors from nude mice (Fig. 6A–C). The knockdown of *LINC00173* in tumors was verified by RT‐qPCR, and the protein levels of RAB1B, PA2G4 and SDF4 were detected by IHC assay (Fig. S5A,B).

To further investigate whether *LINC00173* facilitates the metastasis of NPC cells *in vivo*, we constructed inguinal lymph node metastasis models by plantar subcutaneous injection and lung metastasis models by tail vein injection. As the data showed, the inguinal lymph nodes in the sh0173 group exhibited a diminished size and volume compared with those in the shCtrl group (Fig. 6D–F). H&E staining of foot tumor indicated that the tumors in sh0173 group were less aggressive with impaired invasions into the skin or muscle than were those in the shCtrl group (Fig. 6G). IHC for pan‐cytokeratin of lymph nodes revealed that, compared with the shCtrl group, the sh0173 group exhibited remarkably fewer metastases (Fig. 6H,I). Moreover, in the lung metastasis model, the sh0173 group manifested a smaller nodule on the lung surface (Fig. 6J) and fewer and smaller metastatic nodes in the lung slice visualized by H&E staining (Fig. 6K,L).

## 4 Discussion

Increasing evidence has revealed that lncRNA exert pivotal effects involved in regulating the carcinogenesis and progression of various types of cancers, including NPC [12, 13, 14, 15, 16, 17, 18, 19, 20, 21]. Thus, screening new lncRNA and exploring its function and mechanism have important value for NPC patients. We filtered out a novel lncRNA *LINC00173* from our previous microarray data via bioinformatics analysis, and its high expression was verified in NPC tissues and cell lines. NPC patients with higher expression of *LINC00173* had a poorer prognosis. Knockdown of *LINC00173* markedly restrained NPC cell growth and metastasis *in vitro* and *in vivo*. *LINC00173* enhanced the malignancy of NPC through binding to RAB1B1, which facilitated the secretion of PA2G4 and SDF4 proteins (Fig. 6M), providing potential targets for NPC patients.

Currently, *LINC00173* has been reported to function as a cancer promoting or suppressing factor in different tumors [26, 27, 28, 29, 30]. For instance, *LINC00173* facilitates the progression of triple negative breast cancer by inhibiting *miR‐490‐3p* expression [26]. In addition, it regulates Etk expression by sponging *miR‐218* to promote drug resistance and progression of small cell lung cancer [27], and it promotes angiogenesis and progression of lung squamous cell carcinoma via the regulation of *VEGFA* expression by sponging *miR‐511‐5p* [28]. Conversely, *LINC00173* is reported to be downregulated in cervical cancer, and it inhibits cell proliferation and invasion by regulating the *miR‐182‐5p*/*FBXW7* axis [29]. It can also repress tumor growth by inhibiting the levels of Sphingosine kinase 1 in pancreatic cancer [30]. Interestingly, these studies demonstrate that *LINC00173* regulates aggressive phenotypes of tumors mainly through a ceRNA mechanism. In this study, we uncovered a novel mechanism for *LINC00173*, that is, to facilitate the aggressiveness through binding to RAB1B protein in NPC.

RAB1B is a member of the RAB protein family that are low molecular mass monomeric GTPase [31]. RAB1B functions in the early secretory pathway and is essential for the vesicular trafficking between endoplasmic reticulum (ER) and Golgi apparatus [24, 25]. Rab1b‐GTP can promote the COPII/COPI exchange by recruiting GBF1 at the ER exit site interface and facilitate COPI vesicle formation by inducing GBF1 recruitment at the Golgi complex [32, 33]. RAB1B is also involved in the assembly and secretion of lipoproteins [34]. Recently, it has been reported that RAB1B can be recruited by PITPNC1 to the Golgi apparatus, thus promoting the secretion of pro‐invasive and pro‐angiogenic factors and drive several tumor metastases [35]. On the other hand, RAB1B can be inhibited by *miR‐135a* and promotes cancer cell proliferation and invasion in non‐small cell lung cancer [36]. In this study, we uncovered the strong binding association between *LINC00173* and RAB1B protein through RNA pulldown assay followed by the mass spectrometry, and verified by western blotting and RIP assays. We discovered that *LINC00173* did not affect the levels of mRNA and protein of RAB1B, which prompted us to further explore the mechanism of *LINC00173* in driving NPC growth and metastasis.

For this purpose, SUNE1 cells stably expressing sh0173 and shCtrl were subjected to conditioned culture, and the culture supernatant was collected and sent for mass spectrometry analysis. We filtered out a series of low abundance proteins after *LINC00173* knockdown, which manifested that *LINC00173* could promote the secretion of pro‐proliferative and pro‐invasive proteins in NPC cells. LncRNA have been reported to regulate malignant phenotype of various tumors by affecting the extracellular secretion pathway. For example, *lncRNA‐AK131850* can enhance the secretion of vascular endothelial growth factor in mature osteoclasts, which in turn promotes angiogenesis of endothelial progenitor cells [37], and GPER‐induced *lncRNA‐Glu* can enhance glutamate secretion to promote triple‐negative breast cancer cells invasion and metastasis [38]. Additionally, *lncRNA‐UCA1* can promote pituitary cancer cell growth by enhancing the secretion of prolactin [39].

According to the mass spectrometry analysis of concentrated supernatant, PA2G4 and SDF4 were selected for further investigation in this study. PA2G4 is an RNA‐binding protein involved in growth regulation [40]. PA2G4 can be induced into the nucleus by the pseudogene *PA2G4P4* to inhibit glioblastoma cell apoptosis [41]. It can bind YTHDF2 to increase FYN expression and promote hepatocellular carcinoma metastasis [42]. Moreover, PA2G4 is reported to be regulated by noncoding RNA [43, 44]. For example, *circERBB2* promotes gallbladder cancer progression by regulating PA2G4‐modulated ribosomal DNA transcription [43]. Knockdown of *LINC00987* suppresses acute myeloid leukemia progression by inhibiting the IGF2BP2‐mediated PA2G4 expression [44]. More importantly, the expression of PA2G4 has been reported to be elevated in NPC and is associated with a poor prognosis [45]. On the other hand, SDF4 is a member of the CREC protein family and is involved in regulating the calcium‐dependent cellular activities [46]. SDF4 interacts with CXCR4 to trigger VEGFD expression in endothelial cells and promotes the angiogenesis of lung cancer [47]. In this study, knockdown of *LINC00173* facilitated the secretion of PA2G4 and SDF4 rather than affected their protein levels. After treatment with an exocytosis inhibitor Exo‐1, *LINC00173* knockdown significantly suppressed the secretion of PA2G4 and SDF4. Moreover, RAB1B overexpression reversed the diminished secretory activity caused by *LINC00173* knockdown (Fig. 6M). These results demonstrate that *LINC00173* can promote the malignancy of NPC by driving the secretion of PA2G4 and SDF4.

## 5 Conclusion

In summary, high *LINC00173* expression predicted a poor prognosis in NPC patients, whereas knockdown of *LINC00173* could significantly inhibit NPC cell proliferation and metastasis *in vitro* and *in vivo*. Further mechanism study found that *LINC00173* facilitated the extracellular secretion of PA2G4 and SDF4 proteins, and this exocytosis effect relied on the stimulation of RAB1B. Functional experiments manifested that knockdown of PA2G4 or SDF4 reversed the aggressive phenotypes of NPC cells induced by *LINC00173* overexpression. Our study highlights the prognostic value of *LINC00173* in NPC, and identifies the *LINC00173*‐RAB1B‐PA2G4/SDF4 axis as a potential therapeutic target for NPC patients.

## Conflict of interest

The authors declare no conflict of interest.

## Author contributions

Y‐QL and NL conceived the study and provided scientific direction. S‐WH, Y‐LL, SG, M‐LY, S‐YH and X‐RT performed the experiments. YZ and XL collected the clinical samples, clinical information and follow‐up data. S‐QZ and YZ helped with the data analyses and discussions. S‐WH, Y‐QL and NL analyzed data and wrote the paper. All authors approved the final paper.

## Supporting information


**Fig. S1.** *LINC00173* is identified as a non‐coding RNA.
**Fig. S2.** Relative expression of *LINC00173* after transfected with *LINC00173* knockdown or overexpressing plasmids.
**Fig. S3.** Gene ontology analysis with *LINC00173* specifically pulled down proteins.
**Fig. S4.** Relative expression levels of *LINC00173*, PA2G4 and SDF4 after transfection with indicated plasmids.
**Fig. S5.** The expression levels of *LINC00173*, RAB1B, PA2G4 and SDF4 in tumor tissues.
**Table S1.** Primers for RT‐qPCR, vector construction and shRNA.
**Table S2.** Relationship between *LINC00173* expression and clinicopathologic features of NPC patients (*n* = 214).
**Table S3.** Cox regression analysis of variables contributing to overall, disease‐free and distant metastasis‐free survivals in NPC patients (*n* = 214).
**Table S4.** The top10 proteins found by mass spectrometry analysis in the *LINC00173* RNA pull down.
**Table S5.** Proteins regulated significantly (*B* ≤ 0.05) in SUNE1 shCtrl versus sh0173 cells, as identified by LC–MS/MS (Top10).

## Data Availability

The key raw data of this study have been deposited into the Research Data Deposit (RDD) platform with the identified accession number RDDB2022287766.
