# Supplementary material for: LINC00173 facilitates tumor progression by stimulating RAB1B‐mediated PA2G4 and SDF4 secretion in nasopharyngeal carcinoma
Source: Mol Oncol. 2023 Jan 23;17(3):518–33. doi: 10.1002/1878-0261.13375 (PMC9980309; doi:10.1002/1878-0261.13375)
Supplement: Supplementary file 1 — Fig. S1. LINC00173 is identified as a non‐coding RNA. Fig. S2. Relative expression of LINC00173 after transfected with LINC00173 knockdown or overexpressing plasmids. Fig. S3. Gene ontology analysis with LINC00173 specifically pulled down proteins. Fig. S4. Relative expression levels of LINC00173, PA2G4 and SDF4 after transfection with indicated plasmids. Fig. S5. The expression levels of LINC00173, RAB1B, PA2G4 and SDF4 in tumor tissues. Table S1. Primers for RT‐qPCR, vector construction and shRNA. Table S2. Relationship between LINC00173 expression and clinicopathologic features of NPC patients (n = 214). Table S3. Cox regression analysis of variables contributing to overall, disease‐free and distant metastasis‐free survivals in NPC patients (n = 214). Table S4. The top10 proteins found by mass spectrometry analysis in the LINC00173 RNA pull down. Table S5. Proteins regulated significantly (B ≤ 0.05) in SUNE1 shCtrl versus sh0173 cells, as identified by LC–MS/MS (Top10). [file MOL2-17-518-s001.docx]

**Supplementary information**

***LINC00173* facilitates tumor progression by stimulating RAB1B-mediated PA2G4 and SDF4 secretion in nasopharyngeal carcinoma**

Shi-Wei He^1†^, Ye-Lin Liang^1†^, Yuan Zhang^1†^, Xu Liu^1†^, Sha Gong^1^, Ming-Liang Ye^1^, Sheng-Yan Huang^1^, Xi-Rong Tan^1^, Shi-Qing Zhou^1^, Yin Zhao^1^, Na Liu^1*^, Ying-Qing Li^1*^


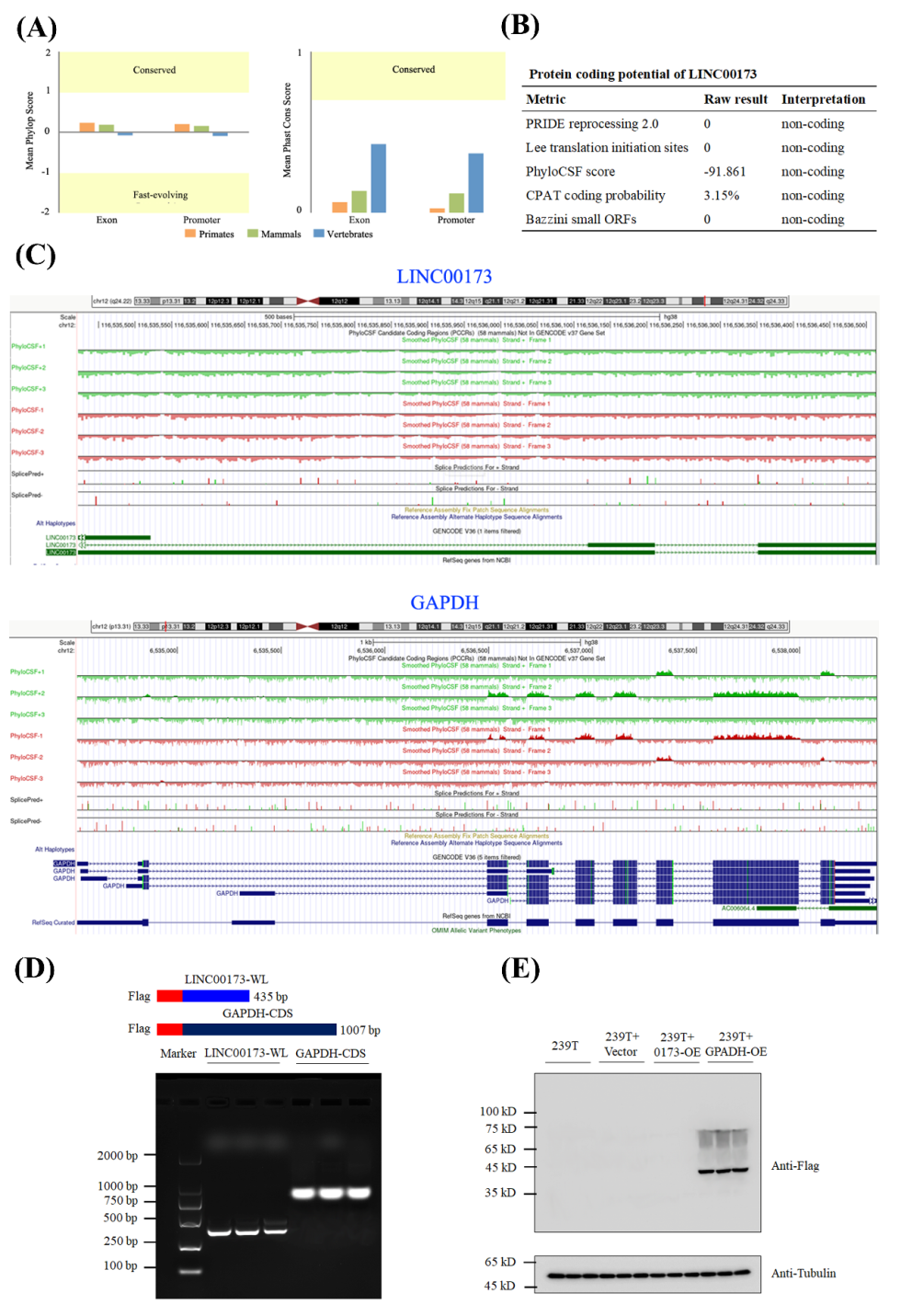


**Fig. S1.** *LINC00173* is identified as a non-coding RNA. (A) AnnoLnc2 was used to predict the evolution of *LINC00173*. (B) LNCipedia database was used to analysis the protein coding potential of *LINC00173*. (C) The PhyloCSF scores were displayed by six frames for each codon. Regions with a score less than 0 were identified as non-coding regions, while greater than 0 were predicted to be coding regions. *GAPDH* was used as a control protein-coding gene. (D) Full length of *LINC00173* or *GAPDH* cDNA was cloned into Flag-tag plasmid (n=3). (E) Western blot (WB) analysis the protein band in 293T cells after transfected with Flag-*LINC00173* or Flag-*GAPDH* plasmid (n=3). Tubulin was used as an internal control.


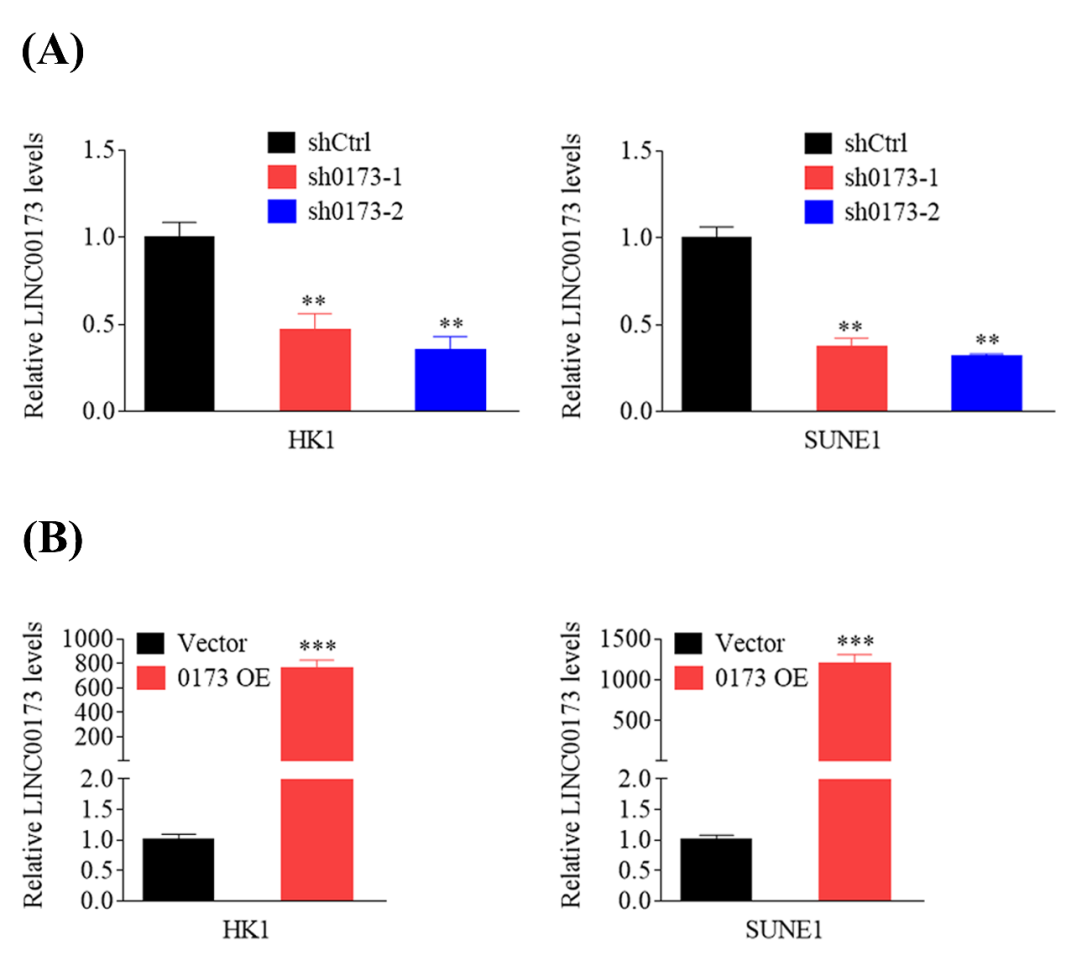


**Fig. S2.** Relative expression of *LINC00173* after transfected with *LINC00173* knockdown or overexpressing plasmids. (A) RT-qPCR assay analysis of *LINC00173* expression in HK1 and SUNE1 transfected with shCtrl or sh*LINC00173* (sh0173) plasmids (n=3). (B) RT-qPCR assay analysis of *LINC00173* expression in HK1 and SUNE1 transfected with vector or *LINC00173* overexpression (0173 OE) plasmids (n=3). Data were presented as mean±SD, and the *p*-values were determined by Student’s *t*-test (***p* < 0.01; ****p* < 0.001).


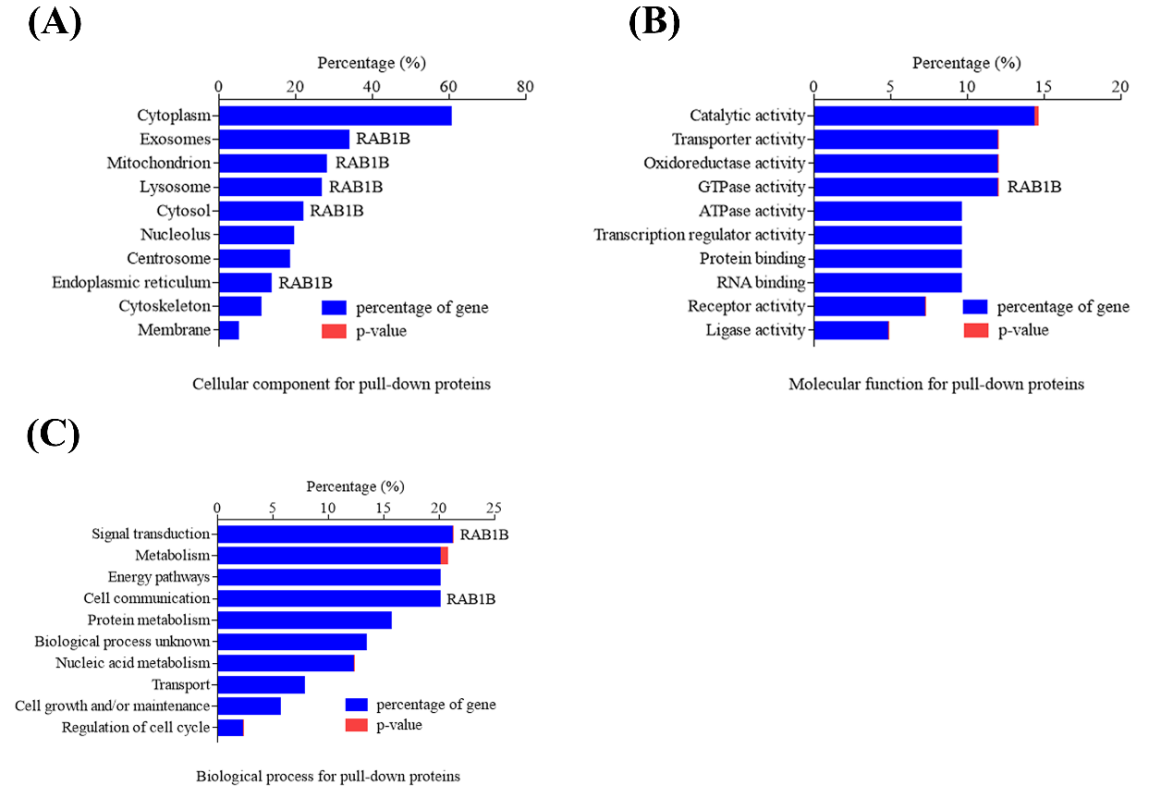


**Fig. S3.** Gene Ontology analysis with *LINC00173* specifically pulled down proteins. (A-C) Cellular component (A), molecular function (B) and biological process (C) analysis with the *LINC00173* specifically pulled down proteins.


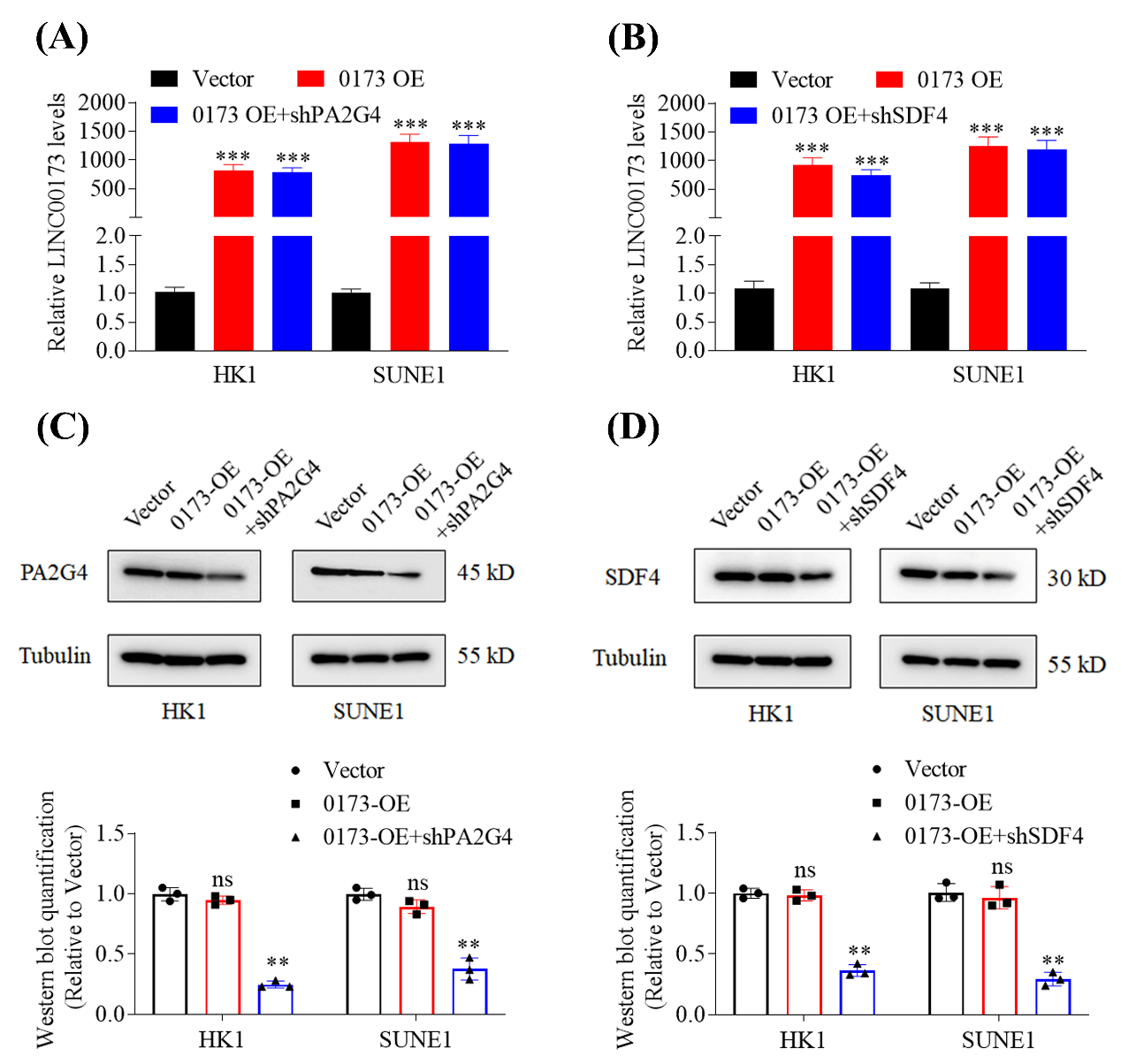


**Fig. S4.** Relative expression levels of *LINC00173*, PA2G4 and SDF4 after transfected with indicated plasmids. (A) RT-qPCR analysis of *LINC00173* expression from HK1 and SUNE1 co-transfected with 0173 OE and sh*PA2G4* plasmid. (B) RT-qPCR analysis of *LINC00173* expression from HK1 and SUNE1 co-transfected with 0173 OE and sh*SDF4* plasmid. (C) Western Blot (WB) analysis and quantification of PA2G4 expression from HK1 and SUNE1 co-transfected with 0173 OE and sh*PA2G4* plasmid. (D) WB analysis and quantification of SDF4 expression from HK1 and SUNE1 co-transfected with 0173 OE and sh*SDF4* plasmid. Tubulin was used as an internal control. Data were presented as mean±SD, and the *p*-value were determined using the Student’s *t*-test (ns, no statistically significant; ***p* < 0.01; ****p* < 0.001).


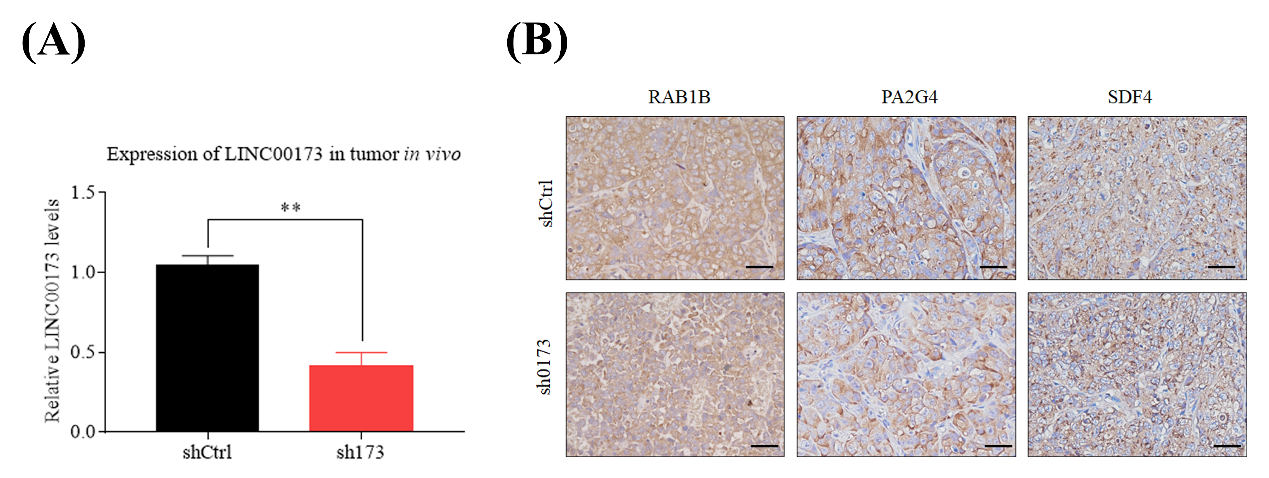


**Fig. S5.** The expression levels of *LINC00173*, RAB1B, PA2G4 and SDF4 in tumor tissues. (A) Relative expression of *LINC00173* in NPC tumor tissues of nude mice. Data was presented as mean±SD, and the *p*-value were determined using the Student’s *t*-test (***p* < 0.01). (B) Immunohistochemistry analysis of the expression of RAB1B, PA2G4 and SDF4 in tumor tissues. Scale bar, 100 μm.

**Table S1.** Primers for RT-qPCR, vector construction and shRNAs

| **Name** | **Sequences (5'-3')** |
| --- | --- |
| **Primers for RT-qPCR with freshly-frozen tissues and cells** | |
| LINC00173 F | ATCGTGAGTGCTCGGGTCAG |
| LINC00173 R | GAATAGGAGTCTCCAGGCAGG |
| RAB1B F | CCATCACTTCCAGCTACTACCG |
| RAB1B R | TCGCTGGCATAGCGGTCAATCT |
| PA2G4 F | GCTCACCTTTGTGCTGAAGCTG |
| PA2G4 R | GCTGCTTCAACTGGTGTGACAG |
| SDF4 F | GGAGAGCAAGACACACTTCCGC |
| SDF4 R | GAGTTCCTCGTTGAGCCTGATG |
| U6 F | CTCGCTTCGGCAGCACA |
| U6 R | AACGCTTCACGAATTTGCGT |
| GAPDH F | GTCTCCTCTGACTTCAACAGCG |
| GAPDH R | ACCACCCTGTTGCTGTAGCCAA |
| **Primers for RT-qPCR with FFPE tissues** | |
| LINC00173 F | ATTCACCCCCTTGTTGAGGC |
| LINC00173 R | ACTCACGATGTGCCAAGGAA |
| GAPDH F | AACGGATTTGGTCGTATTGG |
| GAPDH R | TTGATTTTGGAGGGATCTCG |
| **shRNA sequences** | |
| sh0173-1 | CCTACATGTACTCAGATCACG |
| sh0173-2 | GGAAAGATACCAGATTGATCT |
| shPA2G4-1 | CCACCAGCATTTCGGTAAATA |
| shPA2G4-2 | CCTGGTCGTGACCAAGTATAA |
| shSDF4-1 | GACGAGTATAAGGTGAAGTTT |
| shSDF4-2 | GAACTCAAAGTGGACGAGGAA |
| **Primers for plasmid constructs** | |
| LINC00173 F | CGCGGATCCCGAGGCTCCCACCTGCTCTAA |
| LINC00173 R | CCGCTCGAGTGCCAGAGTGACTGGGAGTTTA |
| RAB1B R | CGACGCGTATGAACCCCGAATATGACTACCTGTTTA |
| RAB1B F | CCCTCGAGCTAGCAACAGCCACCGCCAGCCGGCTT |
| GAPDH F | CGCGGATCCATGGGGAAGGTGAAGGTCGGAGTC |
| GAPDH R | CCGCTCGAGTTACTCCTTGGAGGCCATGTGGGC |
| **Primers for LINC00173 sense and antisense** | |
| 0173 sense F | CGCGGATCCCGAGGCTCCCACCTGCTCTAA |
| 0173 sense R | CCGCTCGAGTGCCAGAGTGACTGGGAGTTTA |
| 0173 anti-sense F | CGCGGATCCTGCCAGAGTGACTGGGAGTTTA |
| 0173 anti-sense R | CCGCTCGAGCGAGGCTCCCACCTGCTCTAA |

**Table S2.** Relationship between *LINC00173* expression and clinicopathologic features of NPC patients (n = 214)

| **Variable** | **LINC00173** | | ***p*-value** |
| --- | --- | --- | --- |
|  | **Low expression (N, %)** | **High expression (N, %)** |  |
| **Age** |  |  |  |
| ≤ 45 years | 53 (49.5) | 48 (44.9) | 0.584 |
| > 45 years | 54 (50.5) | 59 (55.1) |  |
| **Gender** |  |  |  |
| Female | 28 (26.2) | 19 (17.8) | 0.186 |
| Male | 79 (73.8) | 88(82.2) |  |
| **VCA-IgA** |  |  |  |
| < 1:80 | 10 (9.3) | 17 (12.6) | 0.216 |
| ≥ 1:80 | 97 (90.7) | 90 (87.4) |  |
| **EA-IgA** |  |  |  |
| < 1:10 | 19 (17.8) | 23 (21.5) | 0.606 |
| ≥ 1:10 | 88 (82.2) | 84 (78.5) |  |
| **T Stage** |  |  |  |
| T1-T3 | 86 (80.4) | 90 (84.1) | 0.592 |
| T4 | 21 (19.6) | 17 (15.9) |  |
| **N Stage** |  |  |  |
| N0-N1 | 58 (45.8) | 52 (48.6) | 0.494 |
| N2-N3 | 49 (54.2) | 55 (51.4) |  |
| **TNM Stage** |  |  |  |
| III | 68 (63.6) | 68 (63.6) | 0.556 |
| IV | 39 (36.4) | 39 (36.4) |  |

Abbreviations: VCA-IgA, viral capsid antigen immunoglobulin A; EA-IgA, early antigen immunoglobulin A; TNM, tumor-node-metastasis. The *p-*values were determined by χ^2^ tests.

**Table S3.** Cox regression analysis of variables contributing to overall, disease-free and distant metastasis-free survivals in NPC patients (n = 214)

| **Variable** | **Univariate analysis** | | | **Multivariable analysis** | | |
| --- | --- | --- | --- | --- | --- | --- |
|  | **HR** | **95 % CI** | ***p-value*** | **HR** | **95 % CI** | ***p-value*** |
| **Overall survival** |  |  |  |  |  |  |
| LINC00173 level (high vs. low) | 2.18 | 1.30-3.66 | **0.003** | 2.23 | 1.42-3.98 | **0.001** |
| TNM Stage (IV vs. III) | 2.10 | 1.28-3.45 | **0.003** | 2.05 | 1.42-3.97 | **0.001** |
| Age (≥ 45 vs. <45 years) | 1.17 | 0.71-1.91 | 0.540 |  |  |  |
| Gender (Male vs. female) | 0.45 | 0.21-0.96 | **0.039** | 0.44 | 0.21-0.92 | **0.028** |
| VCA-IgA (≥ 1:80 vs. < 1:80) | 0.71 | 0.24-2.10 | 0.530 |  |  |  |
| EA-IgA (≥ 1:10 vs. < 1:10) | 1.14 | 0.45-2.90 | 0.781 |  |  |  |
| **Disease-free survival** |  |  |  |  |  |  |
| LINC00173 level (high vs. low) | 1.69 | 1.01-2.82 | **0.045** | 1.71 | 1.03-2.85 | **0.039** |
| TNM Stage (IV vs. III) | 1.81 | 1.08-3.02 | **0.023** | 1.80 | 1.10-2.95 | **0.020** |
| Age (≥ 45 vs. <45 years) | 1.19 | 0.71-1.98 | 0.510 |  |  |  |
| Gender (Male vs. female) | 0.41 | 0.19-0.92 | 0.029 | 0.398 | 0.18-0.88 | **0.022** |
| VCA-IgA (≥ 1:80 vs. < 1:80) | 0.78 | 0.25-2.43 | 0.664 |  |  |  |
| EA-IgA (≥ 1:10 vs. < 1:10) | 1.19 | 0.46-3.05 | 0.723 |  |  |  |
| **Distant metastasis-free survival** |  |  |  |  |  |  |
| LINC00173 level (high vs. low) | 2.00 | 1.10-3.64 | **0.023** | 2.10 | 1.17-3.79 | **0.013** |
| TNM Stage (IV vs. III) | 1.93 | 1.08-3.46 | **0.026** | 1.87 | 1.07-3.23 | **0.029** |
| Age (≥ 45 vs. <45 years) | 1.09 | 0.61-1.95 | 0.782 |  |  |  |
| Gender (Male vs. female) | 0.48 | 0.20-1.15 | 0.101 |  |  |  |
| VCA-IgA (≥ 1:80 vs. < 1:80) | 0.23 | 0.44-1.19 | 0.080 |  |  |  |
| EA-IgA (≥ 1:10 vs. < 1:10) | 5.26 | 1.03-3.56 | 0.075 |  |  |  |

Abbreviations: TNM, tumor-node-metastasis; VCA-IgA, viral capsid antigen immunoglobulin A; EA-IgA, early antigen immunoglobulin A; HR, hazard ratio. The *p-*values were determined by univariate or multivariate Cox regression analysis. Bold values indicate *p* < 0.05.

**Table S4.** The top10 proteins found by mass spectrometry analysis in the LINC00173 RNA pull down

| Protein | Score | |
| --- | --- | --- |
| HLA class I histocompatibility antigen, B-18 alpha chain, HLA-B | | 304 |
| **Ras-related protein Rab-1B, RAB1B** | | **274** |
| Mitochondrial 2-oxoglutarate/malate carrier protein, SLC25A1 | | 181 |
| Lamina-associated polypeptide 2, isoform alpha, TMPO | | 178 |
| Aspartyl/asparaginyl beta-hydroxylase, ASPH | | 171 |
| Inosine-5'-monophosphate dehydrogenase 2, IMPDH2 | | 167 |
| Mitochondrial import receptor subunit TOM34, TOMM34 | | 165 |
| Putative ubiquitin-conjugating enzyme E2 N-like, UBE2NL | | 162 |
| Glutaredoxin-related protein 5, GLRX5 | | 151 |
| ATP-dependent DNA helicase Q1, RECQL | | 149 |

**Table S5.** Proteins regulated significantly (B ≤ 0.05) in SUNE1 shCtrl versus sh0173 Cells, as Identified by LC−MS/MS (Top10)

| **Protein Name** | **Accession** | **shCtrl-Coverage (%)** | **sh0173-Coverage (%)** | **Ratios(shCtrl/**  **sh0173)** | **-10lgP** | **Functions** |
| --- | --- | --- | --- | --- | --- | --- |
| SDF4 | sp\|Q9BRK5 | 63 | 1 | 63.00 | 238.52 | Calcium dependent cellular activities |
| PA2G4 | sp\|Q9UQ80 | 61 | 1 | 61.00 | 246.73 | Growth regulation |
| CCT5 | sp\|P48643 | 55 | 1 | 55.00 | 248.18 | Folding of proteins upon ATP hydrolysis |
| CACYBP | sp\|Q9HB71 | 70 | 2 | 35.00 | 237.15 | Protein homodimerization activity |
| DDX39B | sp\|Q13838 | 61 | 2 | 30.50 | 271.08 | Mediate ATP hydrolysis during pre-mRNA splicing |
| PSAT1 | sp\|Q9Y617 | 61 | 2 | 30.50 | 217.09 | Carbon metabolism and Vitamin B6 metabolism |
| LOXL2 | sp\|Q9Y4K0 | 29 | 1 | 29.00 | 251.92 | Chromatin binding and electron transfer activity |
| TPI1 | sp\|P60174 | 77 | 3 | 25.67 | 301.37 | Ubiquitin protein ligase binding and triose-phosphate isomerase activity |
| UBA52 | sp\|P62987 | 51 | 2 | 25.50 | 170.83 | Cellular proteins for degradation by the 26S proteosome |
| YBX1 | sp\|P67809 | 71 | 3 | 23.67 | 243.18 | Regulation of transcription and translation, pre-mRNA splicing, DNA reparation and mRNA packaging |

Coverage (%): Ratio of the number of identified effective polypeptide amino acids to the whole protein. -10lgP: Confidence score of peptides.
